# Canada’s Peril

**Published:** 1903-05

**Authors:** 


### EDITORIALS.

### CANADA'S PERIL.

We sincerely trust that the profession in Canada will not allow the school at Montreal to close its doors for want of adequate support. A movement inaugurated by the profession would have a strong bearing upon the Government, through the influence of those engaged in agriculture, dairying, and live-stock breeding, whose interests are greatly imperiled when institutions of this kind are not well supported and properly maintained. The day has gone by when veterinary colleges should be established or continued for individual profit or gain; the day has dawned when these schools should be nurtured only for higher education, a purpose single only to the public welfare and with a constant determination of a true fostering and care of the public health and wealth of all nations.

